# Supplementary material for: Statistical considerations of nonrandom treatment applications reveal region-wide benefits of widespread post-fire restoration action
Source: Nat Commun. 2022 Jun 16;13:3472. doi: 10.1038/s41467-022-31102-z (PMC9203498; doi:10.1038/s41467-022-31102-z)
Supplement: Supplementary file 1 — Supplementary Information [file 41467_2022_31102_MOESM1_ESM.pdf]

Supplementary Information for:
"Statistical consideration of nonrandom treatment applications reveal region-wide benefits of widespread post-fire restoration action"

Allison Simler-Williamson & Matthew Germino

Supplementary Note 1: Details about matching algorithm and post-matching balance of covariates

Nearest neighbor matching of treated and untreated observations on the basis of their propensity scores was completed using the *MatchIt* package in R (Ho, Imai, King, & Stuart, 2011). In *MatchIt*, nearest neighbor matching is conducted by computing a propensity score distance between each treated and each control observation, using logistic regression. Treated observations are then assigned a control match (without replacement) by proceeding through the list of treated units and selecting the closest eligible control unit, based on the propensity score. This algorithm is described as "greedy" because each match is selected one-by-one (beginning with the observations with the highest propensity scores), without considering best fits among the subsequent set of matches or any optimization criteria; however, nearest neighbor matching has been demonstrated to result in similar matched sets and subsequent results as "optimal" matching approaches (see Austin 2013).

We conducted nearest neighbor matching using all biophysical covariates mentioned in the main text. To ensure that covariates would be balanced between treated and untreated groups, we limited the allowable distance between pairs' propensity scores, using a "caliper width" criteria (which determines the maximum allowable difference between paired sites) equivalent to a quarter of the standard deviation of the mean propensity score, as recommended in Guo and Fraser 2010. Caliper modifications to nearest neighbor matching approaches can be effective in eliminating imbalance and reducing bias when discarding of sample units is not an inferential concern (Austin 2013). Unmatched observations (that exceeded the caliper width) were discarded, restricting the sample to a common region of support.

In the unmatched sample, treated and untreated locations differed in their biophysical characteristics, but in the matched subsample, biophysical variables were similar among treated and untreated locations. We diagnosed the effect of propensity score matching (PSM) on balance of the covariates in two ways:

Firstly, we conducted tests for differences in the means for each covariate between treated and untreated groups, using linear and generalized linear models (depending on the structure of the tested variable) containing a single variable for treatment group. Differences in means were identified using the parameter estimate for treatment group. Groups were considered to have statistically important differences in means if the 95% credible intervals for the treatment group parameter did not contain zero. The results of these difference in mean tests are shown below (Supplementary Fig. 2). The full distributions for each variable, before and after PSM, are shown in Supplementary Fig. 3. Before PSM, covariates for each biophysical variable examined differed between treated and untreated groups. Following PSM, there were no nonzero differences between treated and untreated groups for the covariates examined.

Secondly, we examined the effect of each of these biophysical characteristics on the probability of a location being treated, before and after the matching process, shown in Figure 2b in the main text. For the unmatched full sample, the 95% credible intervals for the parameters

associated with each biophysical variable do not include zero, suggesting that these variables have nonzero effects on the probability of a location receiving treatment. Following the matching process, the credible intervals for the effect of each characteristic now included zero (shown in grey in Figure 2b), indicating that in the PSM subsample, these variables no longer had an important effect on the probability of a location receiving treatment. The PSM subsample was limited to a common region of support in terms of probability of receiving restoration treatments (Figure 2a).

Supplementary Figure 1: Results of tests for differences in the means of biophysical covariates between untreated (control) and treated groups (n=20,000 locations before matching, n=11,012 after matching), conducted before and after propensity score matching (PSM), contrasting the full, unmatched dataset with the matched subset. Dots indicate the median posterior parameter estimate, with associated 95% credible intervals (CIs; shown with lines). Group means were considered be statistically different if the 95% CIs for the treatment group parameter did not contain zero (indicated by circular symbols) and similar if 95% CIs contained zero (indicated by triangular symbols). Difference in means tests were conducted using linear regressions (for variables with normal distributions), gamma glms (for positively-bounded, continuous variables), or negative binomial glms (for positively-bounded count variables)

In the full, unmatched dataset, treated and untreated groups differed for all biophysical variables examined. Following PSM, values of covariates were balanced (bottom panel), with no statistically important differences occurring between the treated and untreated groups.

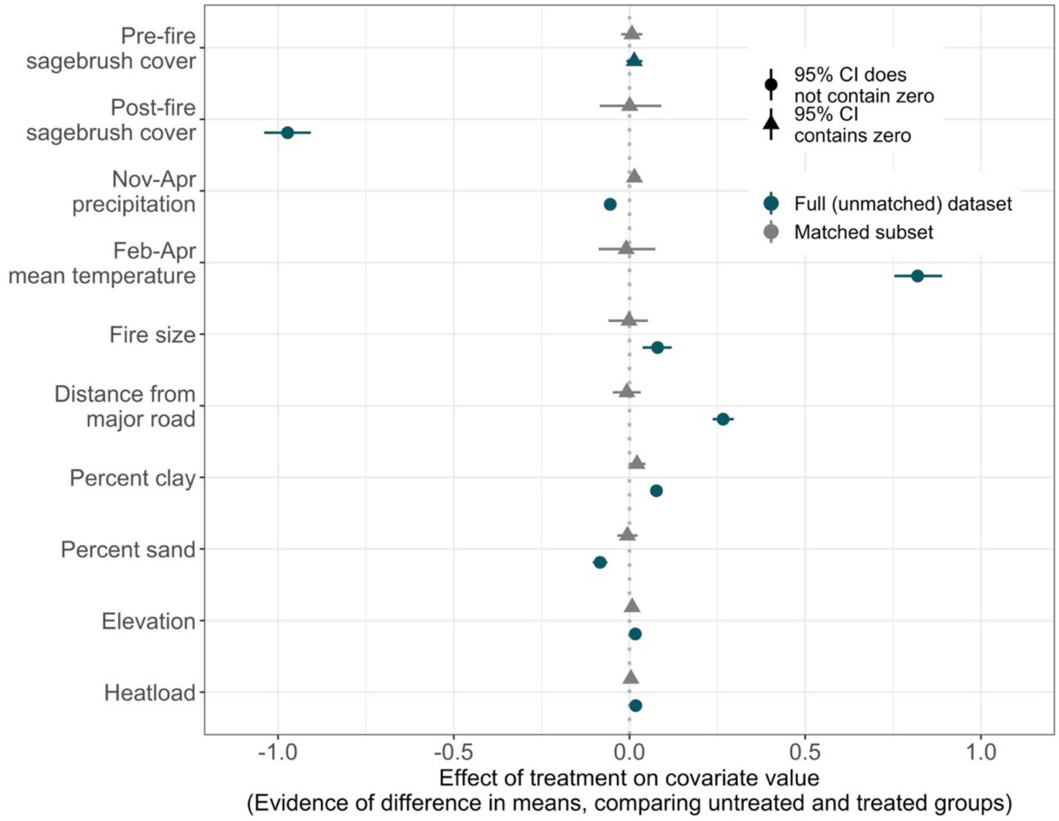

<u>Supplementary Figure 2 (A-F below, G-L on following page)</u>: Comparison of the distribution of biophysical covariates in treated (in red) and untreated (in blue) groups, for the full, unmatched dataset (n=20,000 locations) and the subset remaining after propensity score matching (n=11,012 locations). Smoothed density plots illustrate the full distribution of each covariate. Beneath each set of curves, solid dots indicate the means, and shaded lines represent the 50% (darker line) and 80% quantiles (lighter line) of the distribution. Density plots in some panels were truncated to improve legibility when rare, large values occurred in the tails of each distribution.

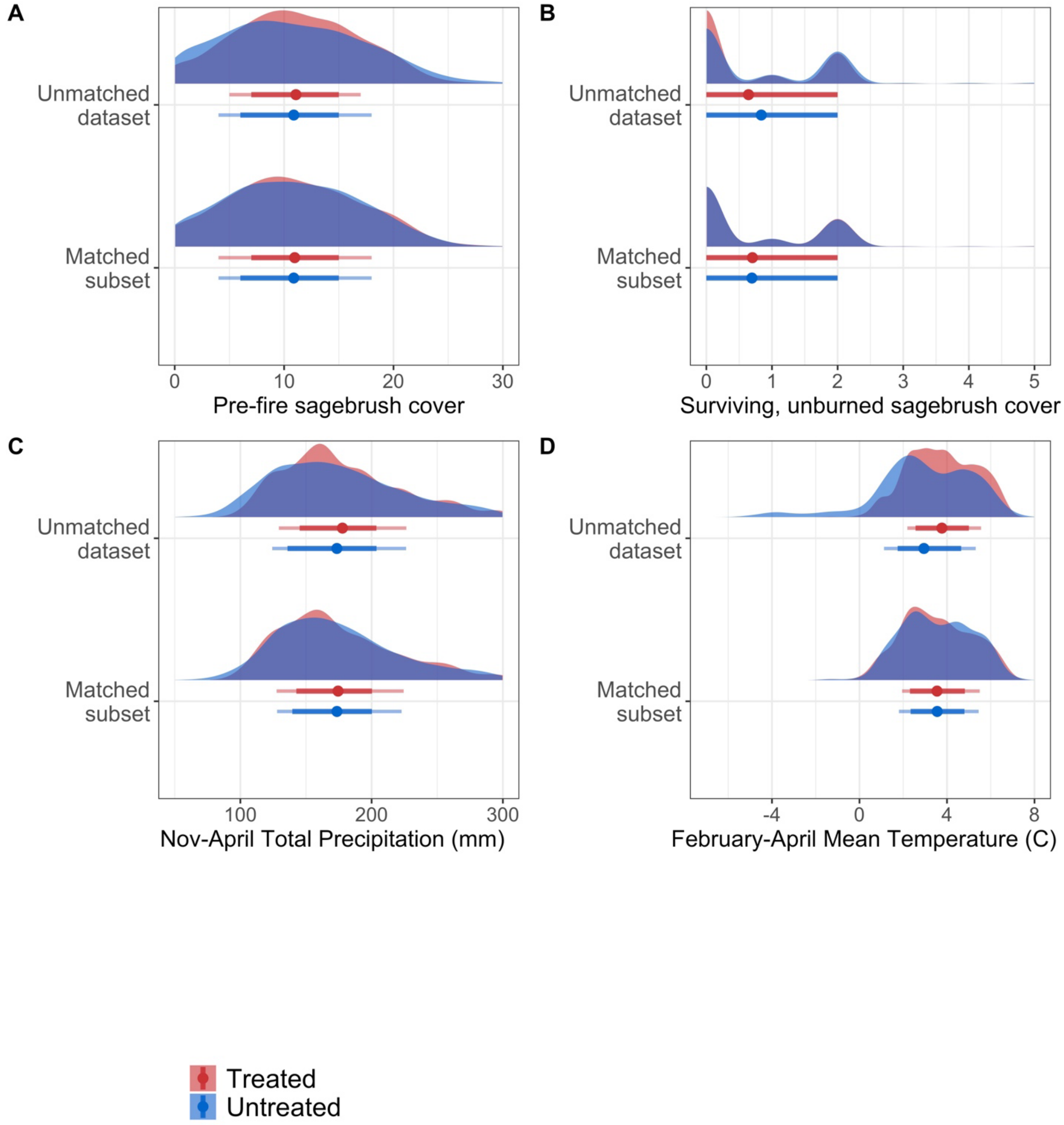

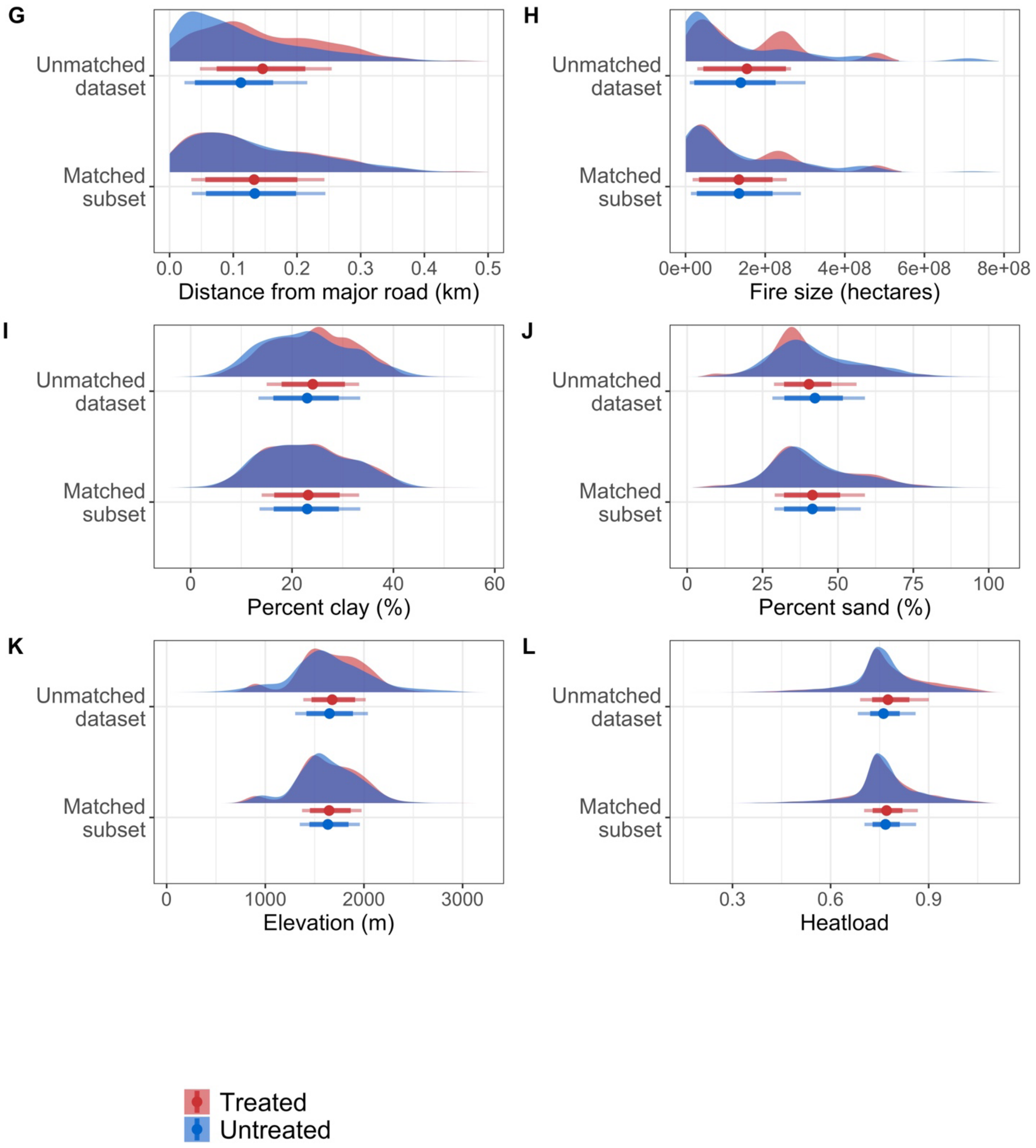

G
Unmatched dataset
Matched subset
0.0
0.1
0.2
0.3
0.4
0.5
Distance from major road (km)
H
Unmatched dataset
Matched subset
0e+00
2e+08
4e+08
6e+08
8e+08
Fire size (hectares)
I
Unmatched dataset
Matched subset
0
20
40
60
Percent clay (%)
J
Unmatched dataset
Matched subset
0
25
50
75
100
Percent sand (%)
K
Unmatched dataset
Matched subset
0
1000
2000
3000
Elevation (m)
L
Unmatched dataset
Matched subset
0.3
0.6
0.9
Heatload
Treated
Untreated

Supplementary Figure 3. A comparison of trends in pre-fire sagebrush cover between pixels that were treated (blue trend) or untreated (red trend) following fire (n=20,000 locations). A key assumption of using difference-in-differences or panel regression approaches is that the treatment groups share similar pre-treatment trends in the response variable of interest. This figure illustrates that, preceding disturbance, subsequently treated and untreated pixels exhibited similar population growth trajectories, despite higher overall mean sagebrush cover in pixels that would later be treated. Lines indicate mean levels of sagebrush cover, with 95% credible intervals, generated using a loess smoothing function.

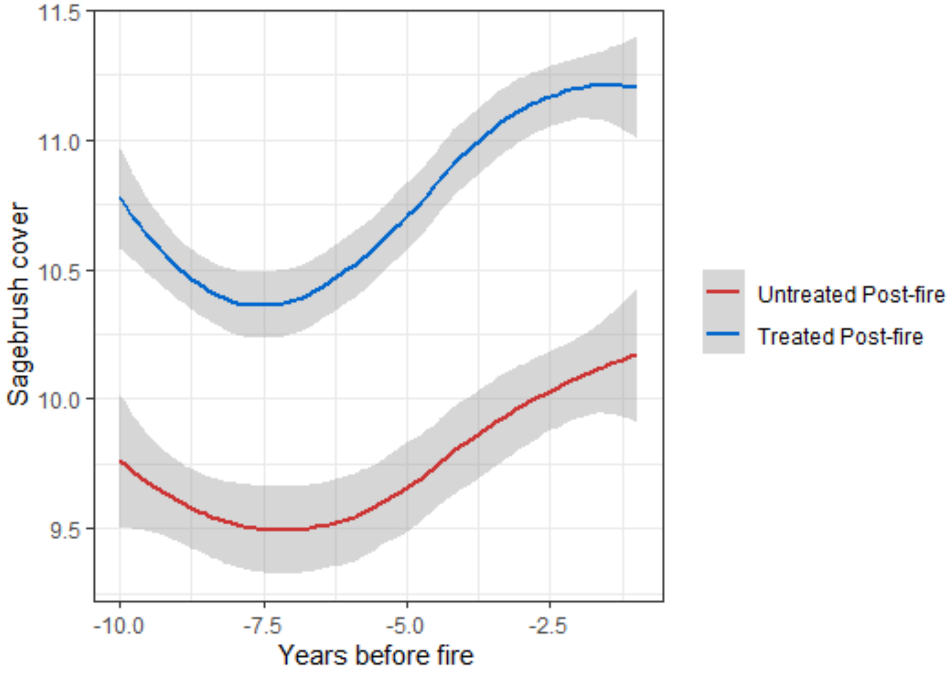

Supplementary Figure 4. Graphical posterior predictive checks for DiD models in the analysis, displaying values of sagebrush cover predicted in posterior draws from the model (yrep), compared to the observed data (y).

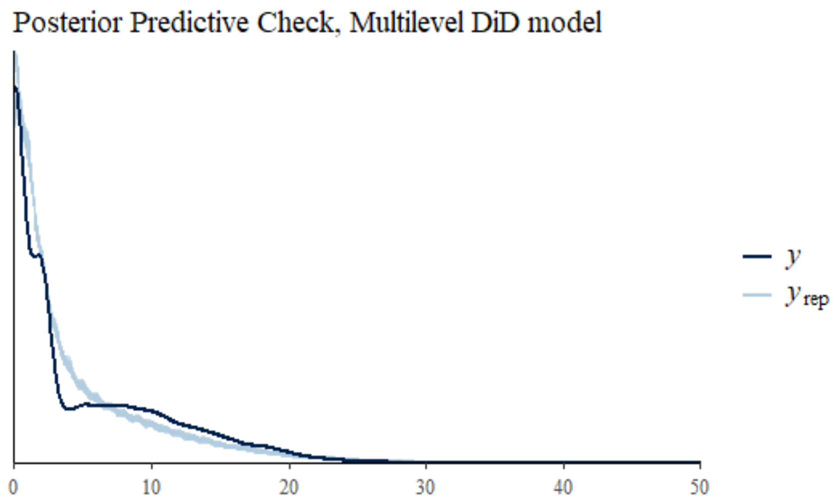

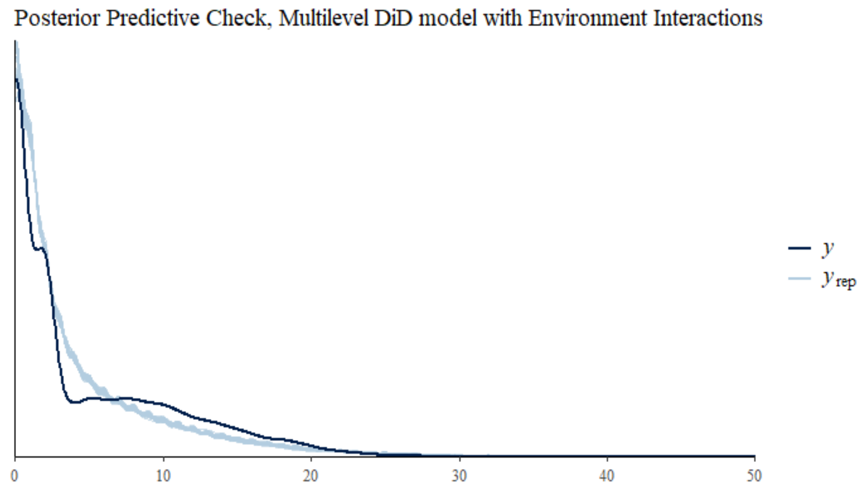

Supplementary Figure 5:. Parameters for indicator variables (including for group identity and treatment occurrence in Panel A, and for time period in Panel B) from comparative treatment effect models (n=20,000 locations before matching; n=11,012 locations after matching). Predicted effects of treatment for treated sites from these models are illustrated in Figure 4 in the main text. Model types are indicated by color. Dots represent median parameter estimates and lines represent the 95% credible intervals (CIs) for the parameter estimate. Some parameters do not appear in all models.

A)

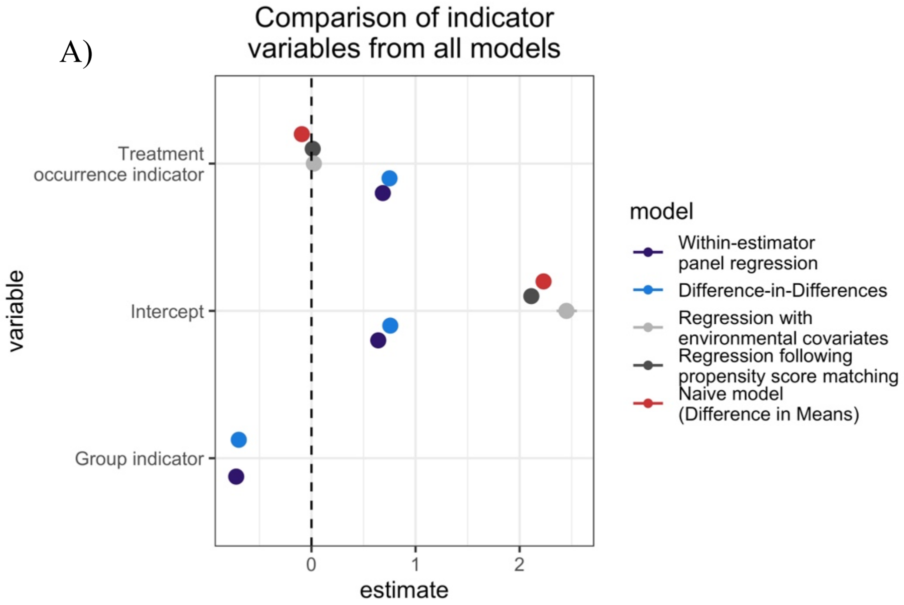

B)

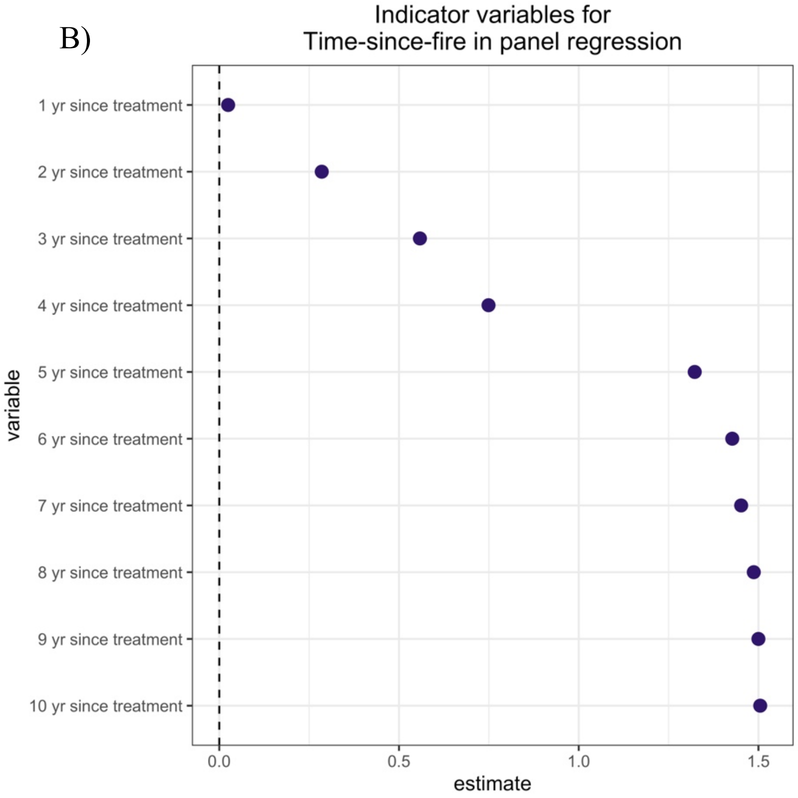

Supplementary Figure 6. Parameters for the effects of environmental covariates on post-treatment sagebrush cover, included in comparative treatment effect models (n=20,000 locations). Predicted effects of treatment for treated sites from these models are illustrated in Figure 4 in the main text. Model types are indicated by color. Dots represent median parameter estimates and lines represent the 50% (thick) and 95% (thin) credible intervals (CIs) for the parameter estimate. Some parameters do not appear in all models.

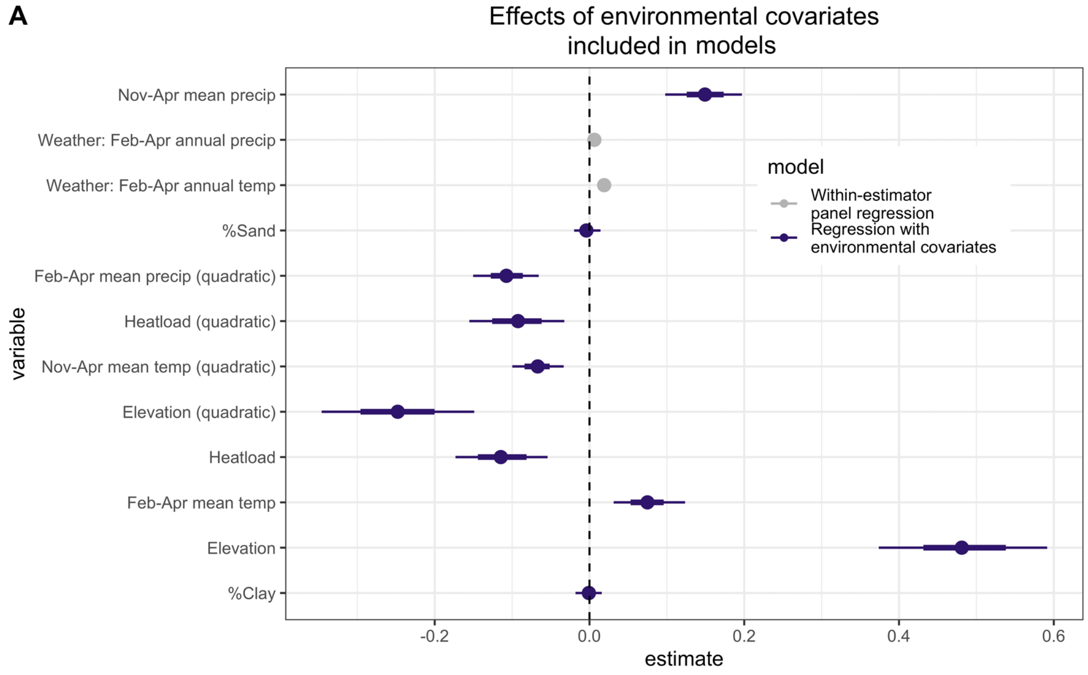

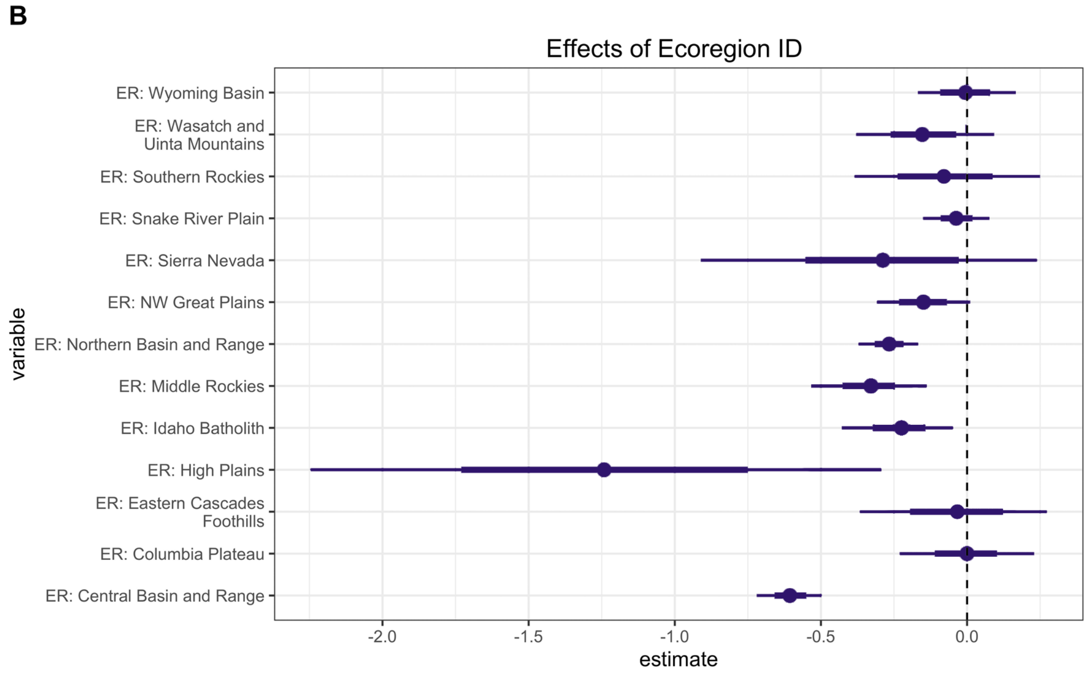

<u>Supplementary Figure 7:</u> Posterior parameter estimates for a difference-in-differences model examining biophysical variation in the treatment effect (indicated by interactions between climate and soil variables and the DID term; n=20,000 locations). Dots represent median parameter estimates and lines represent the 95% credible intervals (CIs) for the parameter estimate.

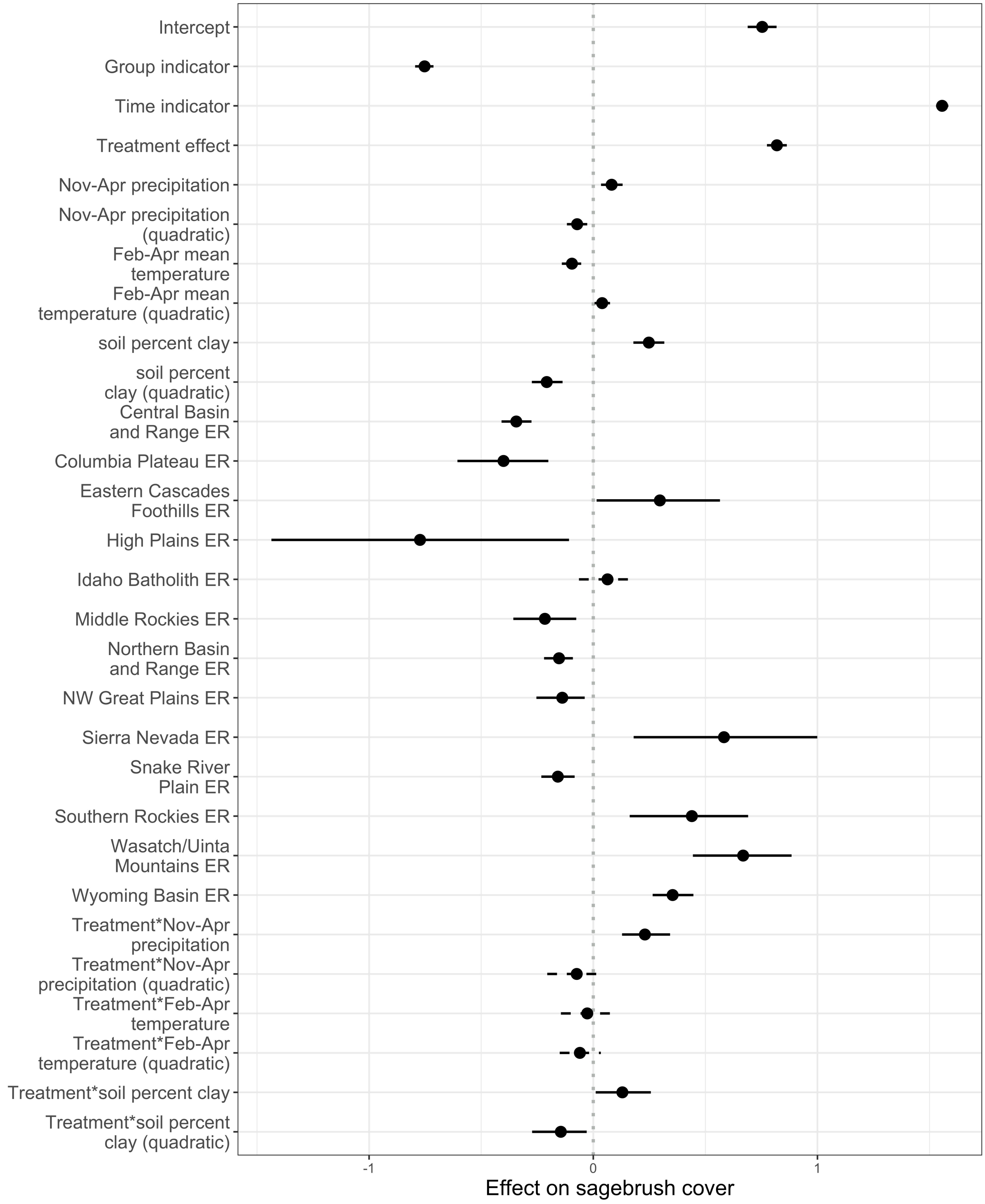

Supplementary Note 2: Assessments of Spatial Autocorrelation
We tested for spatial autocorrelation that was unaccounted for in the multilevel DiD glmm (which contained a varying intercept for fire identity), using a Moran's I correlogram (Supplementary Figure 8) and visual inspection of a spatial variogram (Supplementary Figure 9) to assess whether there were significant sources of unexplained variation associated with the pairwise distances between sites (Supplementary Figure 10). The Moran's I correlogram did not indicate a clear or aggregated pattern between pairwise distances between sites and significant levels of correlation in the normalized residuals from the model (Supplementary Figure 8), and semivariance in the spatial variogram appears to plateau at relatively short distances (~10km, Supplementary Figure 9). These short distances (<10km between locations) represent only 0.4% of the observed pairwise distances in the full dataset (Supplementary Figure 10).

Supplementary Figure 8. Moran's I correlogram assessing spatial autocorrelation in the normalized residuals from the Multilevel DiD model based on pairwise distances between sites. Red dots indicate distances at which there is significant ($p<0.05$; two-sided probability value based on randomization test) correlation between the pairwise distances between sites and their normalized residuals. Correlogram was calculated using the *ncf* package in R (Bjornstad and Cai 2020).

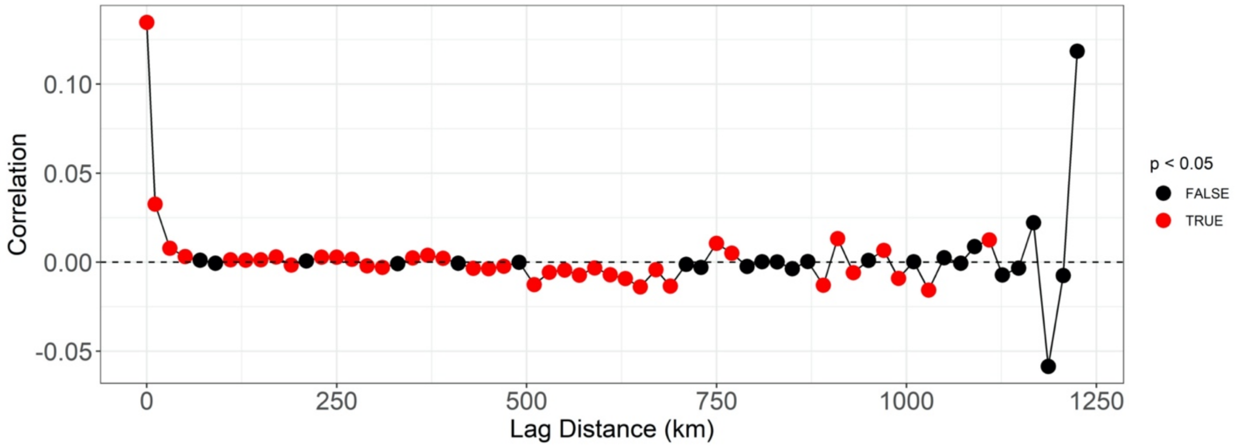

Supplementary Figure 9. Variogram illustrating spatial patterning in the variance of sets of observations, based on pairwise distances between observations. Semivariance was calculated using the *gstat* package in R (Pebesma and Bivand 2005) using the normalized residuals from the multilevel DiD model.

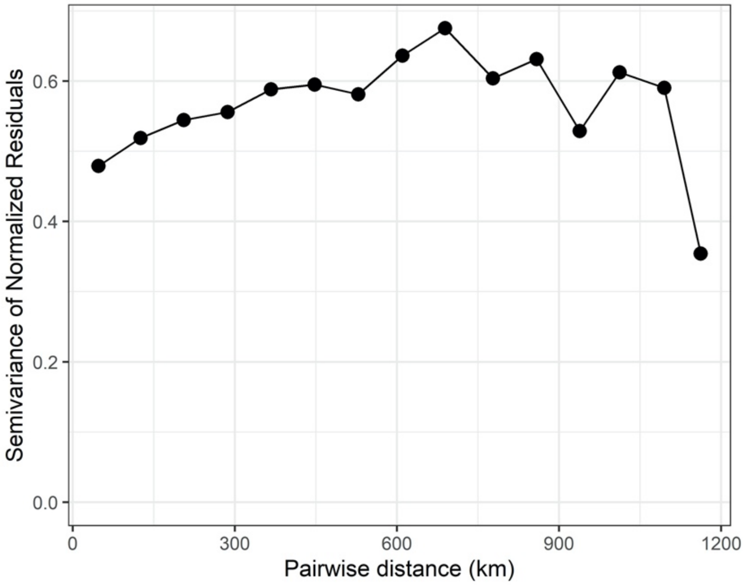

Supplementary Figure 10. The distribution of pairwise distances between randomized sites examined in this study. Pairwise distances less than 10km are shown in black and represent 0.4% of all observations in the dataset.

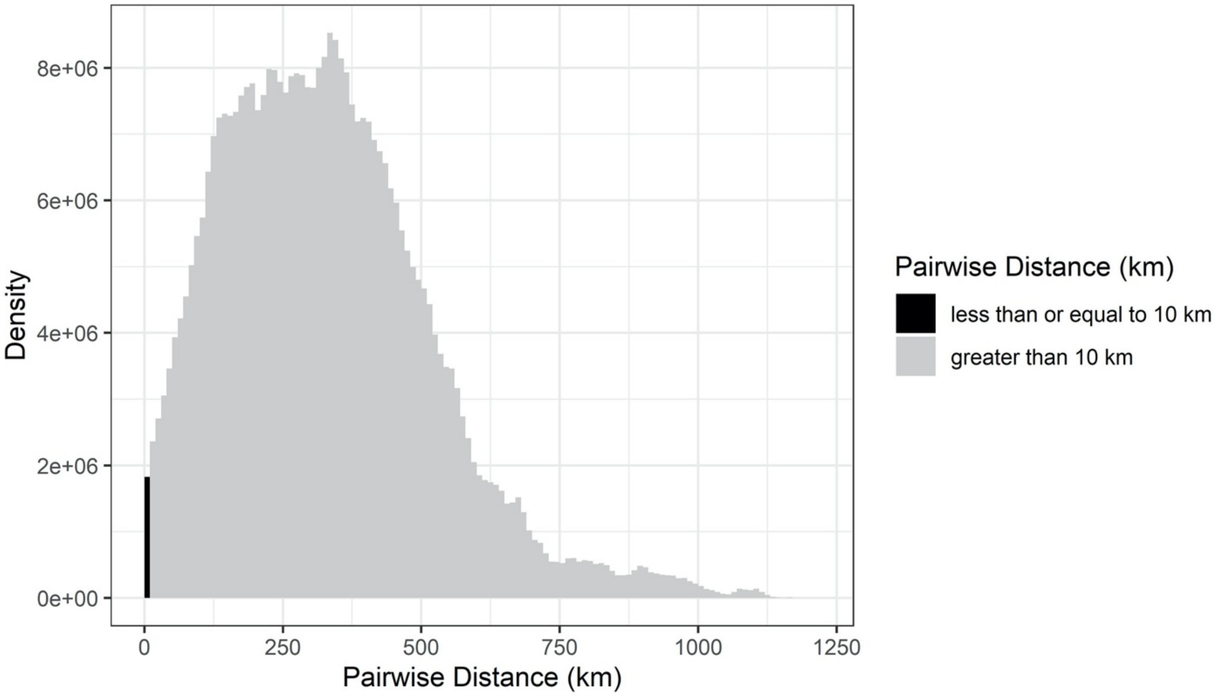

Supplementary Note 3. Propensity Score Matching before Difference-in-Differences

Propensity Score Matching (PSM) is frequently used before conducting DiD analysis (see citations within Daw & Hatfield, 2018). A primary reason for conducting PSM prior to DiD is to improve comparability between groups (i.e., adherence to the assumption of parallel trends between treated and untreated groups). To assess whether this approach altered the conclusions drawn in the analysis presented in the main text, we developed an additional DiD model, analyzing a matched subsample of the full dataset.

Daw and Hatfield (2018) demonstrate that "regression to the mean" can present a threat to inference when conducting PSM prior to DiD estimation. Specifically, they illustrate that matching observations based on time-varying variables, including pre-treatment outcomes, can bias results if pre-treatment outcomes are correlated with treatment assignment. Regression to the mean occurs because, if a selected group contains more extreme values for a given variable (relative to the broader group), subsequent observations for that variable will tend to be closer to the mean. For example, if we measure sagebrush stands and then select a subset of stands with the highest values of sagebrush cover, we expect that same set of stands to have a lower mean that is closer to the broader group mean, if they are remeasured in subsequent years. Because PSM is, in effect, selecting an extreme subset of the dataset for each group (by identifying a region of common support), matching often selects groups that have means that are either higher or lower than the broader group mean. If matching occurs on time-varying covariates, the treated and untreated groups will be likely to "bounce" back toward the true mean of the original, larger pool of observations they were subsetted from. In our analysis, matching based on pre-fire sagebrush cover (given that these variables vary over time and that post-seeding sagebrush cover is our outcome variable) and then conducting DiD could generate bias due to regression to the mean. We expect matching to select a group of untreated locations that have extremely low pre-treatment values of sagebrush cover (to better match treated locations, which had generally lower sagebrush cover; see Figure 2, Figure 3 in main text), which may then regress back to their true group mean, biasing treatment effects toward negative values. Further, DiD already accounts for post-fire surviving sagebrush cover, due to the presence of the "group" variable, which represents the mean value for each group, at the initial timepoint (time=0), before treatment (time*group=0).

Thus, for this comparative analysis, we conducted PSM using the same set of variables described in the main text, except for pre-fire sagebrush cover and surviving/post-fire sagebrush cover, both of which represent time-varying "pre-treatment" outcomes (correlated with treatment application) that could bias DiD estimates. The set of variables incorporated in the revised treatment probability model included: Feb-Apr total precipitation, Feb-Apr total precipitation^2, Feb-Apr mean temperature, Feb-Apr mean temperature^2, elevation, elevation^2, percent soil clay, percent soil sand, Level III Ecoregion, distance from road, distance from road^2, and fire size.

Following this matching process, we retained 13,224 sets of treated and untreated pixels. We then conducted DiD estimation on this matched subset in an identical fashion as described in the text (Table 1). The parameter estimates from the "matched" DiD model are shown below. The estimate for the interaction term (the "incremental treatment effect") shifts slightly with

matching but does not qualitatively differ from the results from the “unmatched” DiD models presented in the main text.

Supplementary Figure 12: Median parameter estimates (dots) from DiD analysis of the matched subsample (n=13,224). 95% credible intervals are depicted but are small enough, relative to the x-axis, to be concealed.

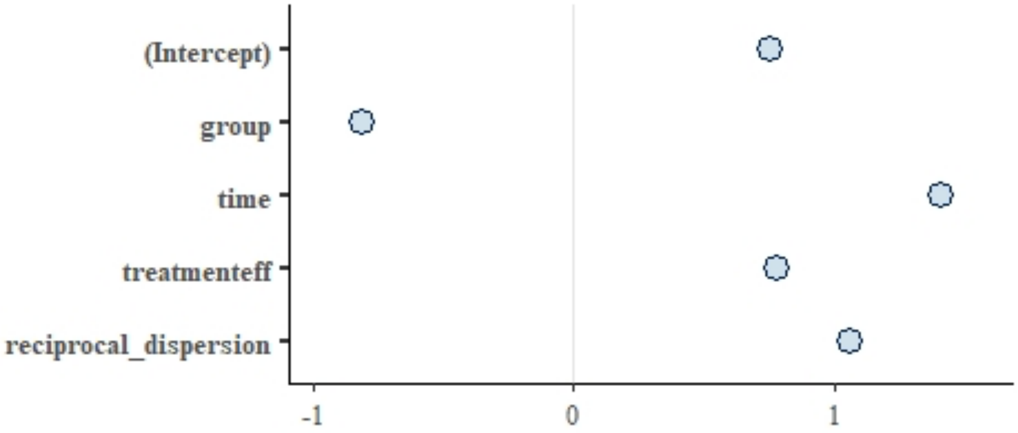

Supplementary Note 4: Comparison of results across different times since fire:
We examined restoration outcomes 10-years following fire to ensure that the stand was beyond the point at which the remotely sensed signal for cover was likely to fluctuate strongly (previously identified as ~ 6 years after fire; Applestein & Germino, 2021), while maximizing the number of observations we could examine within the window for which LTDL data was available to us. To ensure that our results were not sensitive to this selection, we repeated all steps in our analysis (exactly as presented in the main text), examining sagebrush cover 8-years following fire and 15-years following fire. The parameter estimates for each response variable are summarized in Figures 13 and 14 below.

Supplementary Figure 13. Results of the same analysis conducted in the main text, using sagebrush cover 8-years post-fire as the response variable (n= 20000 locations in 1602 fires; subset n = 11102 matched pairs in 1001 fires).

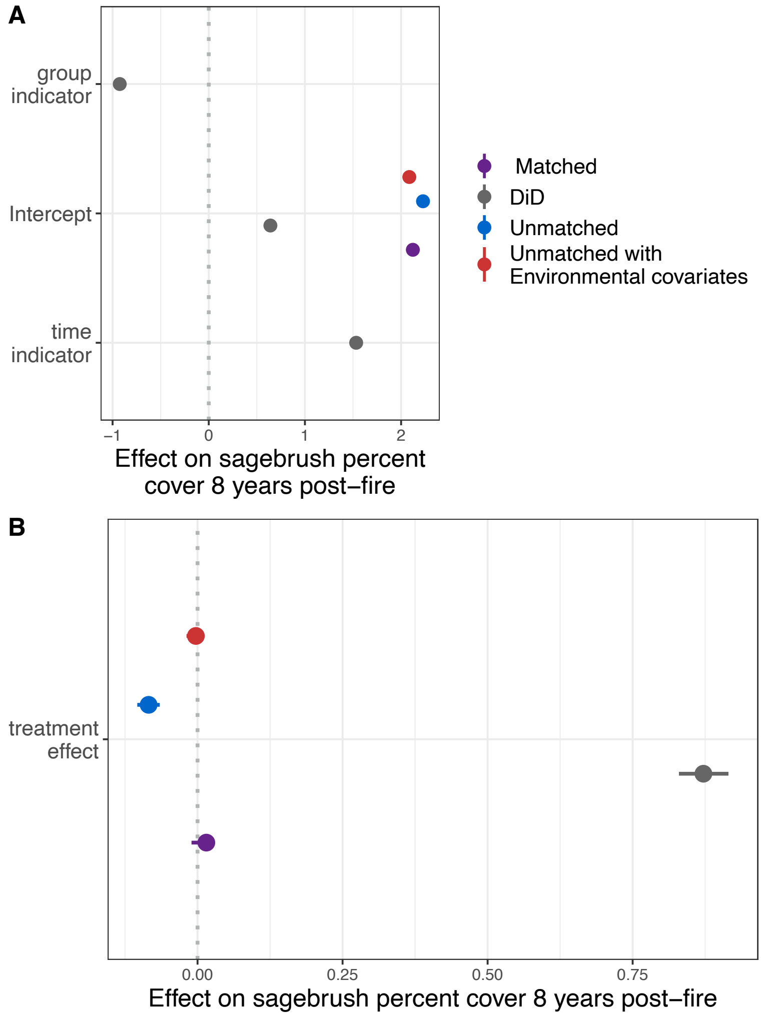

<u>Supplementary Figure 14:</u> Results of the same analysis conducted in the main text, using sagebrush cover 15-years post-fire as the response variable (n= 20000 locations in 1624 fires; subset n = 12408 matched pairs in 1040 fires)

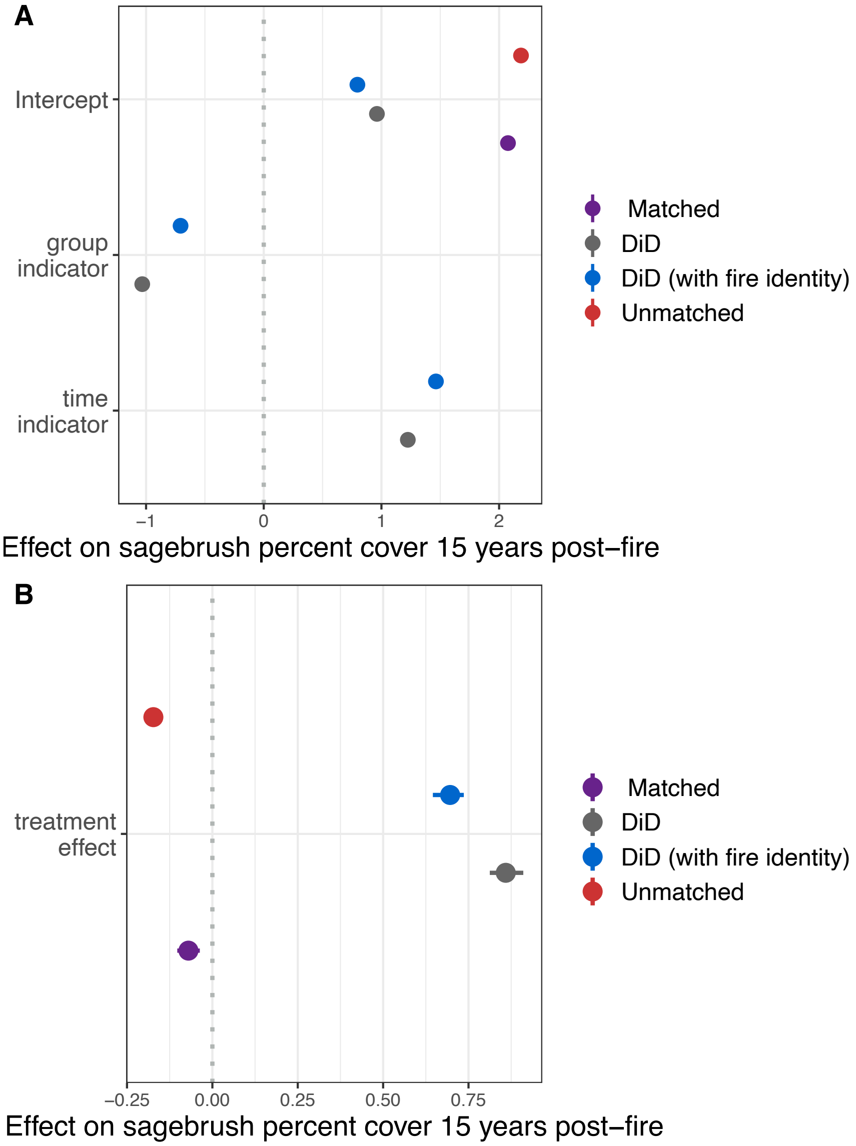

Supplementary Note 5: Additional information about RCMAP remotely sensed product

Sagebrush cover was extracted at 30-m pixel resolution from the USGS Rangeland Condition, Monitoring, Assessment, and Projection product (RCMAP; renamed in the most recent data releases, but formerly known as the National Land Cover Database (NLCD) "Back-in-time" (BIT) products). RCMAP provides estimates of percent cover of functional groups or specific taxa (including sagebrush, annual herbaceous, herbaceous cover, bareground, shrubs, and litter), based on Landsat, Quickbird, and AWiFS imagery for each year from 1985-2018 across the Great Basin (Rigge et al. 2020, 2021, Shi et al. 2020). Based on field validation data, out-of-sample accuracy for the RCMAP shrubland and sagebrush products is ~60% (R^2, Rigge et al. 2020).

The methods used to generate the RCMAP product are described in greater depth in Rigge et al. 2019, 2020, 2021, including additional information about data cleaning, checking, and field-based out-of-sample validation. In short, the product maps fractional cover values using a combination of on-the-ground field data, change detection, and regression tree analysis. The product mapped change in spectral properties from year-to-year using the Landsat archive and a base map. A Landsat image for each year (1985-2018) was selected from spring and from fall, based on cloud, shadow, snow cover, and active fire within the image area. Authors identified a "training" region, in which pixels are unchanged across the time-series from the comparative base map. Then, regression tree models were trained on the unchanged component of the map. For sagebrush and shrub products, there was a specialized training pool for recently burned areas, defined by fire perimeter polygons. Before data releases, estimates were subject to series of post-processing steps to screen for anomalously high or low cover values, based on a set of logic-based rules. Data managers ensured that "successive years of estimates in burned areas have values greater than or equal to the previous year" to avoid anomalous decreases associated with low detection rates (Rigge et al. 2019). In addition, sagebrush cover values were flagged if they exceed a threshold for maximum realistic growth, based on a second-order polynomial regression describing sagebrush population growth with time since fire, informed by values in the literature and from expert knowledge (Rigge et al. 2019). If values are above this limit, they are fixed at the limit. Sagebrush cover is represented as an integer value in the dataset.

The use of satellite-based estimates of vegetation cover involves an inherent tradeoff between spatiotemporal coverage and the measurement error generated by remotely sensed data products. Though extensive field validation studies have been conducted for RCMAP, RCMAP-based estimates may overestimate percent cover measured in the field where sagebrush is scarce (by an estimated 6%, Applestein & Germino, 2021). However, because the DiD approaches adopted here compare the relative changes for repeated measures of a given location and the assumption of parallel trends was fulfilled, we assumed that the comparisons between treated and untreated sites made here are relatively robust to measurement error in RCMAP estimates of sagebrush.

For a full flow chart of all steps involved in production of the RCMAP/NLCD-BIT product, please see Figure 2 in Rigge et al. 2021.

Supplementary Note 6: Comparison of approaches to accounting for clustered observations: Cluster Robust Standard Errors and Multilevel models

Failure to consider clustering of observations can result in artificially small confidence or credible intervals around parameter estimates. To account for the correlation of observations in our dataset, we opted to model the correlation structure directly as part of the model structure, using multilevel models (MLM; also known as hierarchical, random effects, or mixed effects models). This approach has been cited as an appropriate way to account for clustering of observations in panel regression (Wooldridge 2009, Cameron and Miller 2015) that could be more widely used in conjunction with econometric approaches (Oshchepkov and Shirokanova 2022); however, in existing studies that employ difference-in-differences and panel regression approaches, econometric analyses traditionally use cluster robust standard errors (CRSE) as a post-estimation strategy for accounting for clustering structures within datasets (Oshchepkov and Shirokanova 2022).

To demonstrate that our results of our analysis are robust to our choice of modeling approach, we calculated cluster robust standard errors for a frequentist version of our DiD and panel regression models. We used the glm.nb() function in the "MASS" package in R and calculated cluster robust standard errors, using the "sandwich" package in R (Venables and Ripley 2002, Zeileis et al. 2020). We used the vcovCL() option within "sandwich", which obtains multi-way clustered covariance matrix estimators for a variety of model objects, including glm fits. For our DiD and panel regression models, we clustered around either location alone or fire identity and location, to reflect the same clustering factors included in our multilevel model. DiD and panel model structures were identical to those discussed in Table 1 in the main text, except varying intercepts were omitted.

The cluster robust 95% confidence intervals (Supplementary Figure 11) and associated p-values (Supplementary Tables 1 & 2) for the treatment indicator variable (a dummy variable for whether treatments had occurred at the given observation point) indicate a significant effect of the treatment on sagebrush recovery. Though the cluster robust estimates and 95% intervals differ slightly from the multilevel versions of the same models (due to the fact that one approach incorporates the cluster-structure into the model fit, while the other adjusts the standard errors post-estimation), the multilevel and CRSE-based intervals lead to similar conclusions about the efficacy of post-fire seeding efforts, suggesting that our results are robust to our modeling choices.

Supplementary Figure 11: A comparison of estimates for the parameter associated with treatment effect (the treatment group*time period indicator) from DiD and within-estimator panel regression models that account for clustering of observations, either by employing a Bayesian multilevel approach (in blue and purple) or by calculating cluster robust standard errors after fitting a frequentist model (n=20,000 locations). Dots indicate mean estimates, with associated 95% credible or confidence intervals.

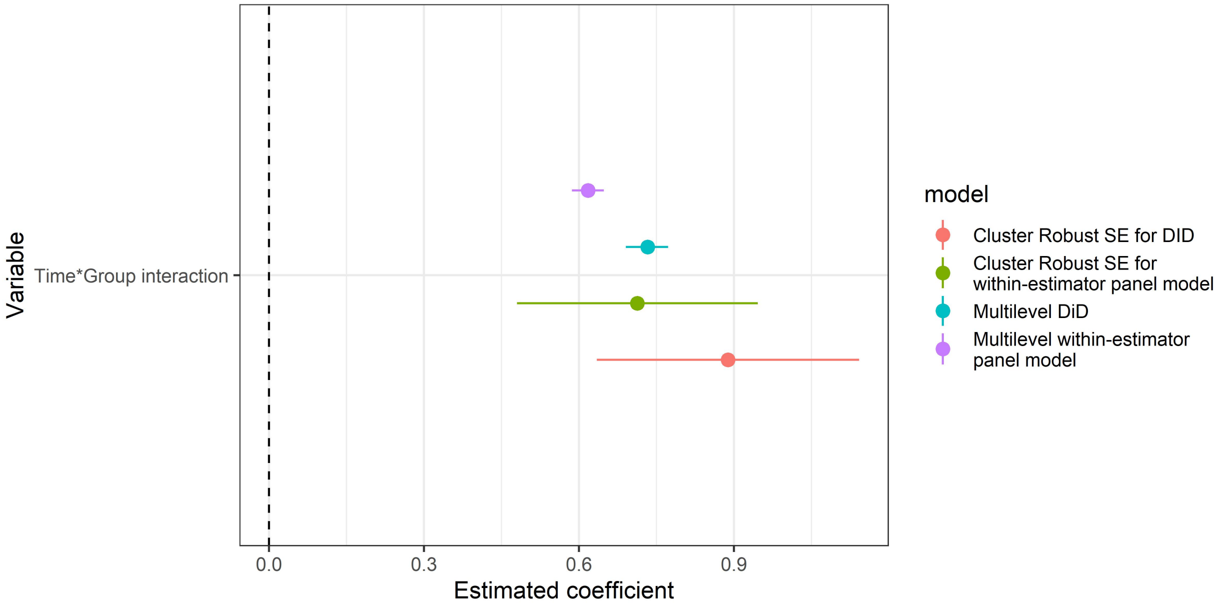

Supplementary Table 1: Parameter estimates from the difference-in-differences regression described above, with standard errors clustered by fire identity and site identity, p-values from two-sided t-tests, and associated 95% confidence intervals. Calculations were performed with the coeftest() function in the lmtest package.

Variable	Mean Estimate	Cluster Robust Standard Error	P-value	Lower bound of 95% CI	Upper bound of 95% CI
Intercept	0.9313	0.0548	9.20566404856797E-65	0.8239	1.0387
Group indicator	-0.9871	0.1406	2.17717674449208E-12	-1.2626	-0.7116
Time indicator	1.2987	0.0467	2.01820585879307E-170	1.2072	1.3902
Group*Time interaction	0.8941	0.1285	3.40008417086506E-12	0.6424	1.1459

Supplementary Table 2: Parameter estimates from the within-estimator panel regression described above, with standard errors clustered by fire identity and site identity, p-values from two-sided t-tests, and associated 95% confidence intervals. Calculations were performed with the coeftest() function in the lmtest package (Zeileis and Hothorn 2002).

Variable	Mean Estimate	Cluster Robust Standard Error	P-value	Lower bound of 95% CI	Upper bound of 95% CI
Intercept	0.9058	0.0524	5.28490529659297 E-67	0.8031	1.0084
Group Indicator	-0.9243	0.1458	2.32471590899654 E-10	-1.2101	-0.6384
Time since Treatment-1	-0.0102	0.0378	0.787006781607875	-0.0843	0.0639
Time since Treatment-2	0.2302	0.0400	8.88011792902373 E-09	0.1518	0.3087
Time since Treatment-3	0.5104	0.0387	1.21754024871945 E-39	0.4345	0.5864
Time since Treatment-4	0.7100	0.0446	3.83950130847201 E-57	0.6227	0.7974
Time since Treatment-5	1.2145	0.0438	5.57435036012979 E-169	1.1285	1.3004
Time since Treatment-6	1.3049	0.0428	9.14624565566131 E-204	1.2209	1.3888
Time since Treatment-7	1.3278	0.0445	4.94999053188788 E-196	1.2407	1.4150
Time since Treatment-8	1.3702	0.0442	3.65432860653278 E-211	1.2836	1.4568
Time since Treatment-9	1.3540	0.0446	1.95514520182072 E-202	1.2665	1.4414
Time since Treatment-10	1.3361	0.0456	4.03748283056883 E-189	1.2468	1.4254
Treatment Indicator	0.7130	0.1189	2.00882327840281 E-09	0.4800	0.9460
Spring total precipitation (scaled)	0.0269	0.0241	0.264097550330154	-0.0203	0.0740
Spring mean temperature (scaled)	-0.1090	0.0282	0.00011003928132163	-0.1642	-0.0538

<u>Supplementary Table 3:</u> Median estimates and associated standard errors for the standard deviations (**σ**) for varying intercept terms included in models in this analysis. Varying intercept estimates are too numerous (n=1,539 for fire identity, n=20,000 for site identity) to display separately.

**Model**	**Group associated with varying intercept**	**Median σ for varying intercept**	**SE(σ)**
Regression containing environmental covariates	Fire identity	0.5271487	0.0005
DID model	Fire identity	0.7557105	0.0003017
	Site identity	0.2854625	0.00015
Within-estimator panel regression	Fire identity	0.6876295	0.00027
	Site identity	0.658275	6.479224e-05

<u>Supplementary References</u>

Applestein, C., and M. J. Germino. 2021. Detecting shrub recovery in sagebrush steppe: comparing Landsat-derived maps with field data on historical wildfires. Fire Ecology 17.

Austin, P.C. 2013. "A Comparison of 12 Algorithms for Matching on the Propensity Score." Statistics in Medicine 33 (6): 1057–69. https://doi.org/10.1002/sim.6004.

Bjornstad, O. N., and J. Cai. 2020. ncf: Spatial Covariance Functions.

Cameron, A. C., and D. L. Miller. 2015. A Practitioner ' s Guide to Cluster-Robust Inference 50:317–372.

Daw, J. R., and L. A. Hatfield. 2018. Matching and Regression to the Mean in Difference-in-Differences Analysis. Health Services Research 53:4138–4156.

Oshchepkov, A., and A. Shirokanova. 2022. Bridging the gap between multilevel modeling and economic methods. Social Science Research in press.

Pebesma, E. J., and R. S. Bivand. 2005. Classes and methods for spatial data in R.

Rigge, M., C. Homer, L. Cleeves, D. K. Meyer, B. Bunde, H. Shi, G. Xian, S. Schell, and M. Bobo. 2020. Quantifying western U.S. rangelands as fractional components with multi-resolution remote sensing and in situ data. Remote Sensing 12:1–26.

Rigge, M., C. Homer, H. Shi, D. K. Meyer, B. Bunde, B. Granneman, K. Postma, P. Danielson, A. Case, and G. Xian. 2021. Rangeland fractional components across the western United States from 1985 to 2018. Remote Sensing 13:1–26.

Rigge, M., H. Shi, C. Homer, P. Danielson, and B. Granneman. 2019. Long-term trajectories of fractional component change in the Northern Great Basin, USA. Ecosphere 10.

Shi, H., C. Homer, M. Rigge, K. Postma, and G. Xian. 2020. Analyzing vegetation change in a

sagebrush ecosystem using long-term field observations and Landsat imagery in Wyoming. Ecosphere 11:1–20.
Venables, W., and B. Ripley. 2002. Modern Applied Statistics with S. 4th Edition. Springer, New York.
Wooldridge, J. M. 2009. Econometrics: Panel Data Methods. Complex Systems in Finance and Econometrics:215–237.
Zeileis, A., S. Köll, and N. Graham. 2020. Various Versatile Variances: An Object-Oriented Implementation of Clustered Covariances in R. Journal of Statistical Software 95:1–36.
Zeileis A, Hothorn T (2002). "Diagnostic Checking in Regression Relationships." *R News*, **2**(3), 7–10. https://CRAN.R-project.org/doc/Rnews/.
