# Supplementary material for: Statistical considerations of nonrandom treatment applications reveal region-wide benefits of widespread post-fire restoration action
Source: Nat Commun. 2022 Jun 16;13:3472. doi: 10.1038/s41467-022-31102-z (PMC9203498; doi:10.1038/s41467-022-31102-z)
Supplement: Supplementary file 3 — Reporting Summary [file 41467_2022_31102_MOESM3_ESM.pdf]

nature portfolio

Corresponding author(s): Allison Simler-Williamson

Last updated by author(s): May 2, 2022

Reporting Summary

Nature Portfolio wishes to improve the reproducibility of the work that we publish. This form provides structure for consistency and transparency in reporting. For further information on Nature Portfolio policies, see our Editorial Policies and the Editorial Policy Checklist.

Statistics

For all statistical analyses, confirm that the following items are present in the figure legend, table legend, main text, or Methods section.

n/a	Confirmed	
☐	☒	The exact sample size (*n*) for each experimental group/condition, given as a discrete number and unit of measurement
☐	☒	A statement on whether measurements were taken from distinct samples or whether the same sample was measured repeatedly
☐	☒	The statistical test(s) used AND whether they are one- or two-sided<br>*Only common tests should be described solely by name; describe more complex techniques in the Methods section.*
☐	☒	A description of all covariates tested
☐	☒	A description of any assumptions or corrections, such as tests of normality and adjustment for multiple comparisons
☐	☒	A full description of the statistical parameters including central tendency (e.g. means) or other basic estimates (e.g. regression coefficient) AND variation (e.g. standard deviation) or associated estimates of uncertainty (e.g. confidence intervals)
☐	☒	For null hypothesis testing, the test statistic (e.g. *F*, *t*, *r*) with confidence intervals, effect sizes, degrees of freedom and *P* value noted<br>*Give P values as exact values whenever suitable.*
☐	☒	For Bayesian analysis, information on the choice of priors and Markov chain Monte Carlo settings
☐	☒	For hierarchical and complex designs, identification of the appropriate level for tests and full reporting of outcomes
☐	☒	Estimates of effect sizes (e.g. Cohen's *d*, Pearson's *r*), indicating how they were calculated

Our web collection on statistics for biologists contains articles on many of the points above.

Software and code

Policy information about availability of computer code

Data collection	We randomly sampled within burned treated and untreated polygons using the spsample() function in the sp package in R (R version 4.1.1; sp package version 1.4.5). The response variable (sagebrush cover) and independent variables for these randomly generated locations were extracted using the raster package in R (Version 3.4.13).
Data analysis	This analysis was conducted using a combination of the brms package (to conduct Bayesian regression analyses; Version 2.16.1) and the Matchit package (to conduct the propensity score matching process; Version 4.2.0) in R version 4.1.1. Weakly informative default priors were used for all regression models, as described in the recommendations generated by the developers of rstan and brms. Plots of the posterior parameter distributions and predictions of marginal effects were supported by tidybayes (Version 3.0.1) and bayesplot packages (Version 1.8.1). Complete details for this analysis can be found in the main text of the manuscript and in the associated Supplementary Information. All code can be found at https://github.com/absimler/nonrandom-seedings.

For manuscripts utilizing custom algorithms or software that are central to the research but not yet described in published literature, software must be made available to editors and reviewers. We strongly encourage code deposition in a community repository (e.g. GitHub). See the Nature Portfolio guidelines for submitting code & software for further information.

Data

Policy information about availability of data

All manuscripts must include a data availability statement. This statement should provide the following information, where applicable:

- Accession codes, unique identifiers, or web links for publicly available datasets
- A description of any restrictions on data availability
- For clinical datasets or third party data, please ensure that the statement adheres to our policy

The locations of treated and untreated burned pixels within sagebrush steppe's range were identified using spatial polygons of past wildfires and past restoration actions (described below) using the Land Treatment Digital Library. Covariates included elevation, soil percent clay and sand, distance from major roads (using the US Census' TIGER product), heat load (calculated from a digital elevation model raster), and spring climate variables, using the grid MET modeled meteorological dataset. The formatted subset of this data that were used in this analysis have been shared via Dryad: https://doi.org/10.25338/B8W63R

Field-specific reporting

Please select the one below that is the best fit for your research. If you are not sure, read the appropriate sections before making your selection.

- [ ] Life sciences
- [ ] Behavioural & social sciences
- [x] Ecological, evolutionary & environmental sciences

For a reference copy of the document with all sections, see nature.com/documents/nr-reporting-summary-flat.pdf

Ecological, evolutionary & environmental sciences study design

All studies must disclose on these points even when the disclosure is negative.

Study description	We conducted a study assessing how the estimation of restoration treatment effects vary, depending on how measured and unmeasured sources of selection bias are incorporated into analysis, using sagebrush steppe ecosystems as a focal system. We compare models that did not consider selection biases to models that integrated propensity score matching (which integrated several measured hypothesized drivers of where restoration treatments may occur), as well as panel regression (Difference in differences) methods that consider unobserved additional drivers of selection bias. We randomly selected 20,000 pixels within sagebrush steppe ecosystems that had either received post-fire restoration treatments (reseeding) or had not, using a combination of remotely sensed estimates of sagebrush cover and a database of restoration actions across the western US. These pixels were nested within >1,500 distinct fire events, and components of our analysis account for this hierarchical structure.
Research sample	The research sample is comprised by remotely sensed estimates of sagebrush density, identified using the RCMAP product, which estimates cover of Artemisia species for the western U.S. Artemisia species are the foundational components of sagebrush steppe ecosystems and have been threatened by a combination of invasion of annual grasses, shifts in fire frequency, and increasing interannual variation in climatic conditions in these ecosystems. This sample is meant to represent the full variability of conditions in recently burned sagebrush steppe ecosystems across the western U.S, across a twenty year period.<br><br>Our analysis also employed spatial polygons of wildfires (using the Monitoring Trends in Burn Severity database) and past restoration actions (using the Land Treatment Digital Library). Covariates included elevation (using the USGS Digital Elevation model raster), distance from major roads (using the US Census' TIGER product), the EPA's Level III ecoregion categories, heat load (calculated according to McCune 2002), and spring climate variables, using the grid MET modeled meteorological dataset.
Sampling strategy	To provide sufficient spatial and temporal coverage across the large region and timeframe examined in this study, we initially randomly sampled 20,000 locations (10,000 treated, 10,000 untreated) within the perimeters of 1,531 fires occurring between 1985-2005 to provide sufficient coverage across sagebrush steppe ecosystems and twenty years of wildfires in the western U.S. This sample size decreased the total number of observations (of paired treated and untreated locations) following the propensity score matching process, which eliminated some observations which did not have corresponding observations with sufficiently similar propensity scores and the opposite treatment status.
Data collection	We did not directly collect any data used in this study. Sagebrush cover estimates were remotely sensed and summarized annually in the existing RCMAP product. The methods used to estimate sagebrush cover remotely are described at length in Homer et al. 2012, 2015 and Rigge et al. 2020, cited in the text. The locations of restoration treatments were identified using the Land Treatment Digital Library (Pilliod & Welty 2013, cited in text), and we selected restoration treatments that included the post-fire seeding of any Artemisia species.<br><br>The lead author identified locations of treated and untreated burned pixels within sagebrush steppe's range using spatial polygons of wildfires (using the Monitoring Trends in Burn Severity database) and past restoration actions (using the Land Treatment Digital Library) using R (Version 4.1.1). She randomly sampled within burned treated and burned untreated polygons using the spsample() function in the sp package in R (Version 1.4.5). The response variable (sagebrush cover) and independent variables for these randomly generated locations were extracted using the raster package in R (Version 3.4.13).
Timing and spatial scale	The timing and spatial scale of this study was defined by the available remotely sensed estimates, which were available to us between 1984-2015.
Data exclusions	We wanted to use pre-fire estimates of sagebrush cover as a covariate for the matching process, so we could not use information about fires that occurred in 1984. We also wanted to model sagebrush cover at a time point that was sufficiently long after the initial

	restoration treatment occurred, to ensure that we were detecting long-term outcomes of restoration actions and to be sure that the remote sensing product was capable of detecting sagebrush. Thus, to allow for us to assess a time point 10 years following the restoration action, we examined fires only up until 2005 (examining outcomes in 2015). The spatial scale of the analysis was defined by the range of sagebrush steppe ecosystems in the U.S. and the extent of the RCMAP product.
Reproducibility	To ensure the reproducibility of this analysis, we have annotated all R code used to conduct this analysis and have published all code via Github (https://github.com/absimler/nonrandom-seedings). We have also shared the locations identified by our random sampling process as a CSV formatted dataset via Dryad (https://doi.org/10.25338/B8W63R). The raw datasets used in this study are widely available via the National Land Cover Database and via the Land Treatment Digital Library.
Randomization	Locations of sagebrush cover estimates were randomly selected using the sp package (Version 1.4.5) in R. We randomly selected 10,000 pixels from within untreated burned areas (using the layers described above) and 10,000 pixels from within treated burned areas. A focal point of this study is the fact that the allocation of these treatments is not random across the landscape; however selection of pixels was random aside from these constraints of treatment and fire history. We considered possible sources of bias in our sequential/comparative analysis.<br><br>The nonindependent/hierarchical structure of this data (i.e. more than one observation within a given fire perimeter) was accounted for by including a multilevel glm for comparison in our analysis.
Blinding	Blinding was not relevant to this study, as the subjects in the study were spatial locations within sagebrush steppe ecosystems and the design was specifically designed to examine the effects of selection biases on estimation of treatment effects.

Did the study involve field work? ☐ Yes ☒ No

Reporting for specific materials, systems and methods

We require information from authors about some types of materials, experimental systems and methods used in many studies. Here, indicate whether each material, system or method listed is relevant to your study. If you are not sure if a list item applies to your research, read the appropriate section before selecting a response.

Materials & experimental systems

n/a	Involved in the study	
☒	☐	Antibodies
☒	☐	Eukaryotic cell lines
☒	☐	Palaeontology and archaeology
☒	☐	Animals and other organisms
☒	☐	Human research participants
☒	☐	Clinical data
☒	☐	Dual use research of concern

Methods

n/a	Involved in the study	
☒	☐	ChIP-seq
☒	☐	Flow cytometry
☒	☐	MRI-based neuroimaging
